# Editorial: Advanced breeding for abiotic stress tolerance in crops

**DOI:** 10.3389/fpls.2023.1265339

**Published:** 2023-08-09

**Authors:** Tianlun Zhao, Xinyang Wu, Meng Jiang

**Affiliations:** ^1^ Hainan Institute, Zhejiang University, Sanya, China; ^2^ The Advanced Seed Institute, Zhejiang University, Hangzhou, China; ^3^ College of Life Sciences, China Jiliang University, Hangzhou, China

**Keywords:** crops, breeding, abiotic stress, gene family, multiomics analysis, mechanism, advanced techniques

## 1 Introduction

Abiotic stress, including extreme temperature, salinity, drought, and other environmental pollution with excessive heavy metals, is regarded to be a global issue in agricultural systems that results in considerable yield and quality losses for crops (Waadt et al., 2022). With the continued rise of the world’s population, it is crucial for sustainable agriculture and food security to develop advanced breeding strategies that effectively mitigate abiotic stress (Zhang et al., 2022). Additionally, a multifaceted strategy is required for crops to enhance their ability to adapt to abiotic stress, including hormone modulation, plant enzymatic system activation, and stress gene expression (Gong et al., 2020). Therefore, understanding how cereal crops react to abiotic stress is crucial (Mittler et al., 2022). Figure out the important characteristics of abiotic stress and their underlying physiological, biochemical, and molecular bases (e.g., genetic, epigenetic, transcriptomic, and metabolomic) will extend our knowledge in breeding efforts to create abiotic stress-resistant crops (Chang et al., 2020).

In this editorial, we set up a Research Topic of Advanced Breeding for Abiotic Stress Tolerance in Crops, which covers up-to-date scientific evidence and the potential for future research to improve our knowledge of the mechanisms that control the development of abiotic stress tolerance in the world’s major crop species. The following themes are included in this Research Topic: (a) Advanced crop breeding applications for increasing abiotic stress resistance in crops; (b) Novel plant growth regulators for enhancing abiotic stress tolerance in plants; (c) Metabolomic and molecular strategies to improve abiotic stress-resistance in crops; (d) Genetic mechanisms related to abiotic stress tolerance in plants and their related traits in plants by quantitative trait loci (QTL) mapping, genome-wide association (GWAS) investigation, or QTL-sequencing; (e) Epigenetic bases of abiotic stress resistance and their applications in crop breeding.

Despite significant advances in understanding the underlying mechanism of abiotic stress, there remain knowledge gaps in these areas, and our Research Topic aims to address these gaps. In the end, we accepted and published 20 articles (16 Original Research, 3 Review, and 1 Perspective) written by 158 researchers from seven different countries, e.g. Australia, United Kingdom, Italy, Greece, Tunisia, India, and China. Unfolded protein response in balancing plant growth and stress tolerance, current trends and insights on EMS mutagenesis application to studies on abiotic stress tolerance and plant development, and achieving abiotic stress tolerance in plants through antioxidative defense mechanisms were reviewed by Liu et al., Chen et al., and Mishra et al. A Perspective named ‘A Holistic and Sustainable Approach Linked to Drought Tolerance of Mediterranean’ was written by Trovato et al. The 16 Original Research articles will be divided into the following four parts for detailed interpretation.

## 2 Assay of gene family under abiotic stress

- Yang et al. included a thorough analysis of 56 *LOR* (*Lurp-One-Related*) genes in *Brassica napus*. Some reports found that *LOR* gene family members acted key functions in defense of *Hyaloperonospora parasitica* (Hpa) in *Arabidopsis*. This research improved our understanding of the *Brassica napus LOR* gene family and may help in the selection and identification of genes for stress-resistant breeding.

- Pang et al. discovered 9 members of the *APX* gene family in the pepper genome according to the conserved domain of the *APX* protein in *Arabidopsis thaliana*. The work discovered the activities of *APX* genes and provided information for further functional characterisation of *CaAPX* genes.

- Zhang et al. identified 104 *NAC* genes in *Camellia sinensis*. The thorough characterisation of *Camellia sinensis NAC* genes provided by the study may operate as a starting point for research into the molecular basis of *CsNACs*-mediated drought response.

- Wang et al. discovered the 65 potential *bZIP* genes in *Lagenaria siceraria* and described their gene structure, phylogenetic and orthologous connections, gene expression patterns in various tissues and cultivars. Through the use of RNA-Seq and RT-PCR, they have examined and confirmed the cold stress-responsive candidate *bZIP* genes in *Lagenaria siceraria*. This has resulted in the discovery of new information regarding the transcriptional control of bottle gourd *bZIP* family genes and their potential roles in the development of cold-tolerant varieties through breeding.

- Yu et al. found that ten *OsSnRK2* genes were discovered in the rice genome distributed across seven chromosomes and grouped into three subfamilies. The results offered valuable details for comprehending the *OsSnRK2* gene family and examining how it responds to drought, salt, and ABA treatment, particularly drought-salt joint stress.

## 3 Multiomics analysis under abiotic stress

- Xu L. et al. employed a genome-wide association study (GWAS) methodology using 90K single nucleotide polymorphisms (SNPs) in a panel of 329 wheat genotypes to identify the quantitative trait loci (QTL) for ARs and RCA. In breeding efforts, a number of genotypes that consistently performed well under various conditions can be utilized to create wheat varieties that can withstand waterlogging.

- Sun et al. discovered that during drought stress, rice plants’ dry matter mass, N content, and N accumulation all grew to varying degrees even when the same amount of N was applied. The findings showed that pathways involved in energy metabolism, such as the phosphotransferase system (PTS) and D-alanine metabolism, were enriched. In general, the work offers a theoretical foundation for enhancing rice’s drought tolerance and N use efficiency.

- Zhao et al. studied the distribution of Zhe-Maidong (*Ophiopogon japonicus*) roots reacting to Cd stress and the features of Cd transformation by growing Zhe-Maidong seedlings for 90 days in Cd-contaminated and uncontaminated soil. The findings may be useful in determining the features of Cd accumulation in Zhe-Maidong and may also serve as a bioinformatic starting point for research on gene functions and the screening of potential Cd-accumulation-related candidate genes.

- Gu et al. discovered that the rice gene *OsSEH1*, which codes for nucleoporins, is a positive regulator for cold stress. The findings showed that wild type plants and osseh1 lines had hypersensitive phenotypes, indicating that *OsSEH1* may influence cold tolerance *via* controlling ABA levels.

## 4 Exploration of underlying mechanism under abiotic stress

- Zhang et al. reported that OsWRKY76 positively controlled rice drought stress. Dehydration stress, exogenous MeJA, and PEG treatments can induce the expression of *OsWRKY76*. The findings provided a new lead in the quest to understand the processes behind drought tolerance by suggesting that OsWRKY76 provides drought tolerance through OsbHLH148-mediated jasmonate signaling in rice.

- Xu J. et al. discovered that the oil-tolerant plant *Mirabilis jalapa* contained a *mannanase* (*MAN*) gene called *MirMAN*, which was cloned and heterologously produced in *Arabidopsis*. The study showed that *MirMAN* is a potentially significant target gene that may be further used to enhance plant tolerance to abiotic stress.

- Zhou et al. investigated the effects of high temperature on rice (*Oryza sativa*) starch metabolite and the higher reductions in *BEI*, *BEIIa*, *BEIIb*, and *SSIVb* expression when exposed to high temperature. The study found that by generating more starch-lipid complexes under high temperatures, the increased long chains of amylopectin and lipids may be the main cause of the enhanced RS content in mutants.

- Su et al. measured physiological-biochemical parameters and assessed the drought tolerance of various wheat cultivars. While demonstrating the possibility of *TaPRX-2A* overexpression in boosting drought tolerance during crop development efforts, the study offered insights into the processes of tolerance.

- Jiang et al. found that during the progressive drought and water recovery phases, the midday transpiration rate Tr (Tr_m_) and daily transpiration (E) as well as plant growth of water recovery at θcri (WR_θcri) was slightly lower or comparable to those of well irrigation (CK), but those of water recovery under severe drought (WR_SD) were significantly lower than those of CK during water stress and did not recover after rehydration. According to the findings, irrigation balances strawberry growth and water use at a key soil water content.

## 5 Advanced techniques in abiotic stress research

- Shen et al. discovered that precisely introducing a regulatory element into a target gene’s 5’UTR might effectively boost the protein abundance of that gene in rice. The research offered a fresh method to control protein translation for crop breeding by demonstrating the viability of such in-locus editing to improve protein expression.

- Si et al. found that virus-induced gene silencing (VIGS) is continuously tracked by a visible gland in cotton. The enhanced VIGS technology will provide another tool for the quick functional identification of many genomes’ undiscovered genes.

## 6 Prospect

It is crucial to comprehend how general and stress-specific response pathways interact since plants must deal with numerous abiotic stresses at once in their natural habitat. Similar to this, stomatal closure and ROS generation are examples of regulatory nodes at which plant responses to abiotic stimuli may also converge. These numerous reactions, as well as the crosstalk between them, maybe simultaneously triggered and result in additive, synergistic, or antagonistic effects, which could improve or impair stress resistance. In order to discover essential molecular targets for breeding stress-resistant crops in the field, it will be necessary to untangle plant responses to various stresses at the molecular level by using multiomics analysis and advanced techniques.

## Author contributions

TZ: Formal Analysis, Software, Supervision, Writing – original draft. XW: Data curation, Funding acquisition, Investigation, Project administration, Writing – original draft. MJ: Conceptualization, Funding acquisition, Methodology, Validation, Writing – review & editing.
